# Identification of Capsular Polysaccharide Synthesis Loci Determining Bacteriophage Susceptibility in Tetragenococcus halophilus

**DOI:** 10.1128/spectrum.00385-23

**Published:** 2023-05-08

**Authors:** Takura Wakinaka, Minenosuke Matsutani, Jun Watanabe, Yoshinobu Mogi, Masafumi Tokuoka, Akihiro Ohnishi

**Affiliations:** a Manufacturing Division, Yamasa Corporation, Choshi, Japan; b NODAI Genome Research Center, Tokyo University of Agriculture, Tokyo, Japan; c Faculty of Food and Agricultural Sciences, Fukushima University, Fukushima, Japan; d Institute of Fermentation Sciences, Fukushima University, Fukushima, Japan; e Department of Fermentation Science, Faculty of Applied Biosciences, Tokyo University of Agriculture, Tokyo, Japan; University of Pittsburgh School of Medicine

**Keywords:** bacteriophage, host receptor, capsular polysaccharide, depolymerase, *Tetragenococcus halophilus*

## Abstract

Bacteriophages infecting Tetragenococcus halophilus, a halophilic lactic acid bacterium, have been a major industrial concern due to their detrimental effects on the quality of food products. Previously characterized tetragenococcal phages displayed narrow host ranges, but there is little information on these mechanisms. Here, we revealed the host’s determinant factors for phage susceptibility using two virulent phages, phiYA5_2 and phiYG2_4, that infect T. halophilus YA5 and YG2, respectively. Phage-resistant derivatives were obtained from these host strains, and mutations were found at the capsular polysaccharide (CPS) synthesis (*cps*) loci. Quantification analysis verified that capsular polysaccharide production by the *cps* derivatives from YG2 was impaired. Transmission electron microscopy observation confirmed the presence of filamentous structures outside the cell walls of YG2 and their absence in the *cps* derivatives of YG2. Phage adsorption assays revealed that phiYG2_4 adsorbed to YG2 but not its *cps* derivatives, which suggests that the capsular polysaccharide of YG2 is the specific receptor for phiYG2_4. Interestingly, phiYA5_2 adsorbed and infected *cps* derivatives of YG2, although neither adsorption to nor infection of the parental strain YG2 by phiYA5_2 was observed. The plaque-surrounding halos formed by phiYA5_2 implied the presence of the virion-associated depolymerase that degrades the capsular polysaccharide of YA5. These results indicated that the capsular polysaccharide is a physical barrier rather than a binding receptor for phiYA5_2 and that phiYA5_2 specifically overcomes the capsular polysaccharide of YA5. Thus, it is suggested that tetragenococcal phages utilize CPSs as binding receptors and/or degrade CPSs to approach host cells.

**IMPORTANCE** *T. halophilus* is a halophilic lactic acid bacterium that contributes to the fermentation processes for various salted foods. Bacteriophage infections of *T*. *halophilus* have been a major industrial problem causing fermentation failures. Here, we identified the *cps* loci in *T*. *halophilus* as genetic determinants of phage susceptibility. The structural diversity of the capsular polysaccharide is responsible for the narrow host ranges of tetragenococcal phages. The information provided here could facilitate future studies on tetragenococcal phages and the development of efficient methods to prevent bacteriophage infections.

## INTRODUCTION

Tetragenococcus halophilus, a Gram-positive halophilic lactic acid bacterium, plays an important role in the fermentation processes for a variety of salted and fermented foods such as salted fish, vegetable pickles, and soy sauce (1–3). Bacteriophages infecting T. halophilus can impede lactic acid fermentation and decrease the quality of food products (4–6). Tetragenococcal phages typically display narrow host ranges, but the genetic background of this phenotypic characteristic has been poorly studied thus far. To develop efficient strategies against bacteriophage infections, the mechanisms of tetragenococcal host-phage interactions must be understood in detail.

The first step of phage infection is the highly specific attachment to binding receptors on the host cell surface. Some phage receptors of Gram-positive bacteria have been identified, such as peptidoglycan, membrane-associated proteins, wall teichoic acid (WTA), and lipoteichoic acid (LTA) (7–9). The presence of specific receptor molecules is necessary for phage infections, which largely dictates the host ranges of the respective phages. Tetragenococcal phage receptors have not been well characterized. We recently identified ribitol-containing WTA as a receptor for *T. halophilus* phage phiWJ7, but another cell wall component was presumed to be involved in phage adsorption since phiWJ7 and other phages could still adsorb to the strains lacking ribitol-containing WTA (6).

The cell walls of lactic acid bacteria frequently contain diverse polysaccharides in addition to peptidoglycan and WTA (10). Such polysaccharides can be divided into three major groups, capsular polysaccharides (CPSs), exopolysaccharides (EPSs), and cell wall polysaccharides (CWPSs); note that this grouping is not strictly defined. CPSs are covalently attached to peptidoglycan and form a capsule around the bacterium. EPSs are secreted into the environment and are loosely associated with the cells. CWPSs are attached to the cell wall but do not form a capsule. These polysaccharides can also interact with phages. For instance, a CWPS of Lactococcus lactis was identified as a receptor for some phages (11), whereas EPSs of L. lactis can act as a physical barrier against phage infection and protect bacteria (12).

The majority of CPSs are produced via the so-called Wzx/Wzy-dependent pathway (13). In this pathway, the synthesis of a repeat unit occurs in the cytoplasm. Next, the repeat units are translocated to the outer side of the cell membrane by the Wzx flippase. The Wzy polymerase connects the repeat units and extends the sugar chain. The mature CPSs are finally attached to peptidoglycan. The gene clusters for the production of CPSs, known as *cap*/*cps* loci, have been described in many Gram-positive bacteria, including lactic acid bacteria (14, 15). Despite numerous structural and compositional varieties of CPSs, the overall organization and core genes of the *cap*/*cps* loci are well conserved in species and are similar even beyond genera. Streptococcus pneumoniae, in which CPS structures and synthesis genes have been best studied, conserves *cpsABCD* as the first four genes of the *cps* locus (16). *cpsABCD* are implicated in CPS regulation and posttranslational synthesis modulation (17), and the region immediately downstream contains genes encoding glycosyltransferases, acyltransferases, and other modifying enzymes; nucleotide sugar synthesis enzymes; the Wzx flippase; and the Wzy polymerase. The genes located downstream of *cpsABCD* are highly diverse among strains and thereby generate a variety of CPS patterns in S. pneumoniae. The repeating units of CPSs in S. pneumoniae have 2 to 8 saccharide residues and are often decorated with *O*-acetyl, pyruvyl, and phosphoglycerol substitutions (18). More than 100 serotypes were distinguished based on the structures of CPSs in S. pneumoniae (19). Many other Gram-positive bacteria possess genes homologous to *cpsABCD* as the core genes of the *cap*/*cps* loci.

The purposes of this study were to clarify the genes determining phage susceptibilities in *T. halophilus*, especially the genes affecting adsorption by phages, and to reveal the reason for the narrow host ranges of tetragenococcal phages. As a result, we discovered the *cps* loci responsible for CPS synthesis and demonstrated that mutations in the *cps* loci can change the phage susceptibilities of *T. halophilus*. Moreover, it is suggested that tetragenococcal phages utilize CPSs as binding receptors and/or degrade CPSs to approach other receptors. These findings will serve as an important basis for future studies on tetragenococcal host-phage interactions and for the development of industrial countermeasures against bacteriophage infections.

## RESULTS

### Generation of phage-resistant derivatives and detection of mutational sites.

To investigate the genes determining phage susceptibility in *T. halophilus*, we attempted the acquisition of spontaneous phage-resistant derivatives and the determination of their mutated sites. Two host strains, YA5 and YG2, and phages infecting these strains were mainly used in this study (Table 1). Phage-resistant derivatives derived from these strains were selected as colonies growing in the presence of the phages, and the mutation sites of the derivatives were clarified by whole-genome sequence analysis. A YA5 derivative resistant to phage phiYA5 was obtained, which we refer to as YA5R1 (see Fig. S1A in the supplemental material). Genome mapping analysis revealed no mutation sites in YA5R1 (data not shown). The mutation responsible for the phenotypic changes in YA5R1 might have occurred in a region missing from the draft genome. Unfortunately, we could not determine the mutation site, and we did not conduct further investigations because the adsorption of phiYA5 to YA5R1 was not impaired, which shows that YA5R1 does not have a mutation involved in phage adsorption (Fig. S1B). Intriguingly, another phage, phiYA5_2, formed larger plaques on YA5R1 than on YA5 (Fig. S1A). Subsequently, a YA5R1 derivative resistant to phage phiYA5_2 was obtained, which we refer to as YA5R1R1 (Fig. S1A). YA5R1R1 contained only one mutation: a transposition of an insertion sequence (IS) in an open reading frame (ORF) encoding a putative polysaccharide pyruvyltransferase (locus tag YA5_022780) (Fig. S2 and Table S1). Another host strain, YG2, is susceptible to phage phiYG2_4, and the phiYG2_4-resistant derivative YG2R1 was obtained from YG2 (see Fig. 3). YG2R1 contained an 8-bp deletion outside ORFs (744 bp upstream of YG2_08150) and a transposed IS in an ORF encoding α-phosphoglucomutase (YG2_16120) (Fig. S3 and Table S1).

**TABLE 1:** Bacterial strains and bacteriophages mainly used in this study

Strain or bacteriophage	Description	Reference or source
Tetragenococcus halophilus strains		
YA5	Sensitive to phiYA5 and phiYA5_2	22
YA5R1	phiYA5-resistant derivative of YA5	This study
YA5R1R1	phiYA5_2-resistant derivative of YA5R1	This study
YA5R2 (YA5_pyruvylTrfase::IS)	phiYA5_2-resistant derivative of YA5	This study
YG2	Sensitive to phiYG2_4	25
YG2R1 (YG2_pgm::IS)	phiYG2_4-resistant derivative of YG2	This study
YG2R2 (YG2_wzy::IS)	phiYG2_4-resistant derivative of YG2	This study
YG2R48 (YG2_acylTrfase::IS)	phiYG2_4-resistant derivative of YG2	This study
Bacteriophages		
phiYA5	Lytic for YA5	Isolated from soy sauce mash
phiYA5_2	Lytic for YA5; *Rountreeviridae*	Isolated from soy sauce mash
phiYG2_4	Lytic for YG2	Isolated from soy sauce mash

### Identification of capsular polysaccharide synthesis loci.

**FIG 1:**
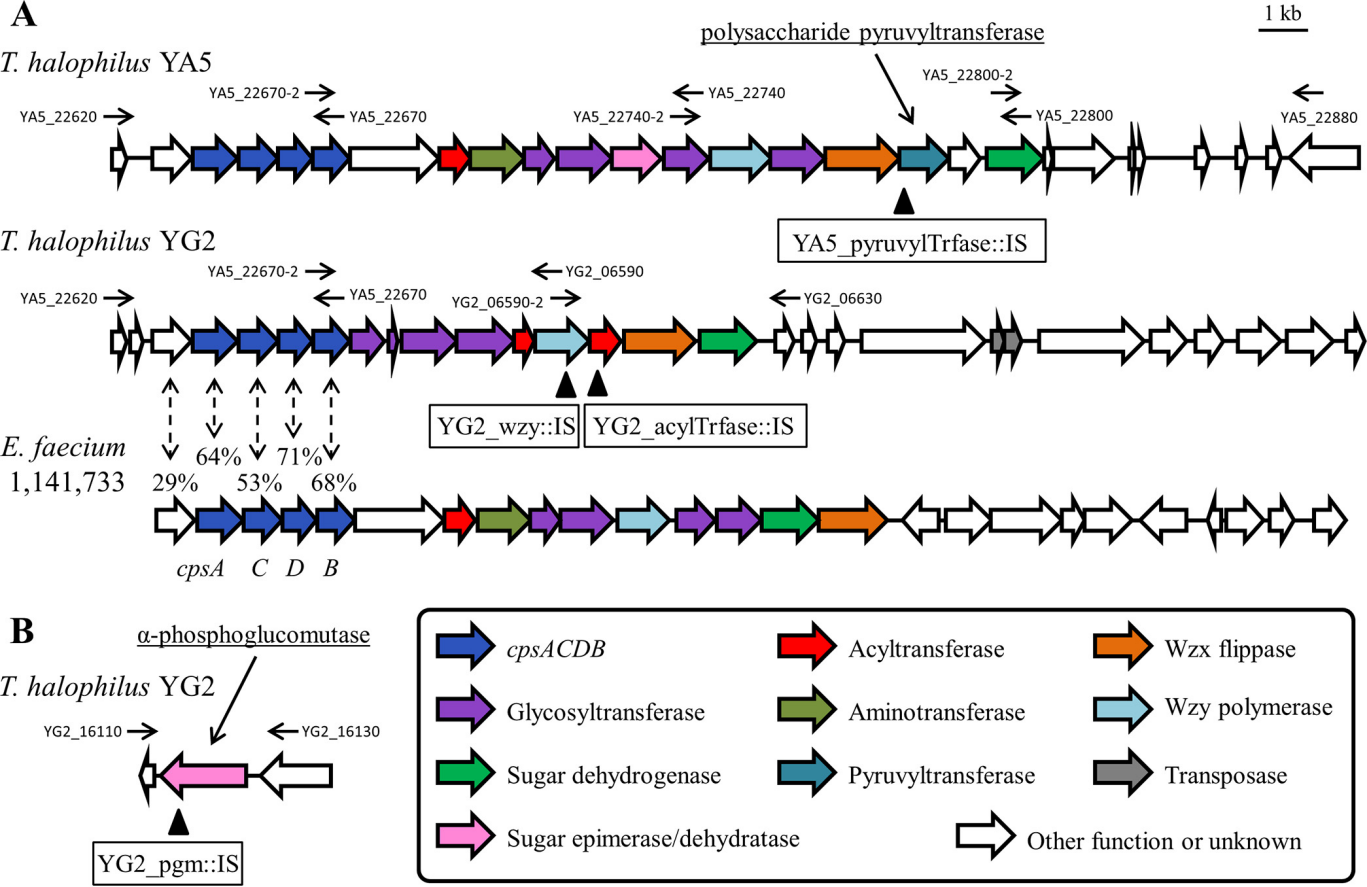
Schematic representation of CPS synthesis genes in *T. halophilus*. The black arrows above ORFs represent the positions at which the primers were designed. The locations of ISs transposed in each derivative are indicated by black triangles below ORFs. (A) Comparison of *cps* loci in *T. halophilus* YA5 and YG2 and E. faecium 1,141,733. The percentages show the amino acid identities between *T. halophilus* and E. faecium. (B) Schematic representation of the α-phosphoglucomutase gene in YG2.

The gene mutated in YA5R1R1 (putative polysaccharide pyruvyltransferase) clustered with genes similar to *cpsACDB* of Enterococcus faecium, the core genes of CPS synthesis (*cps*) loci (Fig. 1A). The *cps* locus of E. faecium contains *cpsACDB* and an unnamed gene adjacent to *cpsA*. These five genes are well conserved in E. faecium and are flanked by genes that are nonhomologous among strains (20). A comparison of the *cps* loci of YA5 and YG2 and those of six *T. halophilus* strains whose complete genomes had been deposited in the DDBJ database revealed that *T. halophilus* also conserves the five genes upstream of the variable regions that contain genes for various glycosyltransferases, modifying enzymes, sugar precursor synthesis enzymes, a repeat unit flippase (Wzx), and a polysaccharide polymerase (Wzy) (Fig. 1A and Fig. S4). Generally, genetic variation in *cps* loci can create different CPS patterns (16), which implies structural differences in CPSs between YA5 and YG2. We extracted CPSs from YA5 and YG2 and analyzed their sugar composition by high-performance liquid chromatography (HPLC) (Fig. S5). Only glucose was detected in the hydrolysates from both strains, but they should contain other types of components such as amino sugars, uronic acids, sialic acids, and sugar alcohols. The polysaccharide pyruvyltransferase of YA5 is presumed to be one of the modifying enzymes for CPS synthesis, indicating that the mutation might have affected the structure and/or expression of CPSs. The gene that contained a transposed IS in YG2R1 (α-phosphoglucomutase) was not located in the *cps* locus, but α-phosphoglucomutase contributes to the generation of sugar precursors such as UDP-glucose and UDP-glucuronic acid for CPS synthesis (21). Based on these results, we hypothesized that CPSs of *T. halophilus* would be involved in the interactions with phages.

### Screening of additional *cps* derivatives.

Unfortunately, a feasible transformation technique for *T. halophilus* has not been established thus far (6), which did not allow us to use a general gene deletion-and-complementation strategy for the analysis of gene function. However, intrinsic ISs of *T. halophilus* jump within the genomes quite actively, and the transposition of ISs can be detected by comparing the lengths of the PCR-amplified DNA fragments covering the targeted genes between the derivative and parental strains (6, 22). Supposing that a deletion or structural alteration of CPSs is responsible for the altered phage susceptibility in the derivatives, other additional phage-resistant derivatives with ISs transposed into the *cps* loci are expected to be found easily. Hence, we obtained additional phiYA5_2-resistant derivatives from YA5 and phiYG2_4-resistant derivatives from YG2 and amplified their DNA regions covering the *cps* loci and the α-phosphoglucomutase gene with the primers shown in Fig. 1 and Table 2. As expected, 16 of 31 phiYA5_2-resistant derivatives showed elongated PCR products covering a part of the *cps* locus (data not shown), and they were named YA5R2 to YA5R17. DNA sequence analyses revealed that all 16 derivatives contained ISs on the polysaccharide pyruvyltransferase gene, similar to YA5R1R1 (Fig. S2 and Table S1). For YG2, among approximately 700 phiYG2_4-resistant derivatives, 41 derivatives had ISs on α-phosphoglucomutase, the same as YG2R1; two ISs, IS*Teha7* and IS*Teha8*, were identified as being active for the first time here (Fig. S3 and S6 and Tables S1 and S2). In addition, one derivative had an IS on the *wzy* polymerase gene (YG2_06590), and six derivatives had ISs on a putative acyltransferase gene (YG2_06600) (Fig. S7 and Table S1). The additional phiYG2_4-resistant derivatives were named YG2R2 to YG2R49. The successful acquisition of additional *cps* derivatives from both host strains strongly suggests the involvement of *cps* loci in host-phage interactions in *T. halophilus*. We picked four derivatives containing an IS in each gene and used them for further investigation. Here, we refer to these selected derivatives as YA5_pyruvylTrfase::IS (YA5R2), YG2_pgm::IS (YG2R1), YG2_wzy::IS (YG2R2), and YG2_acylTrfase::IS (YG2R48) (Fig. 1 and Table 1). Under laboratory conditions, YG2_pgm::IS and YG2_acylTrfase::IS grew as fast as the parental strain YG2 (Fig. S8A). The growth of YG2_wzy::IS was delayed, possibly because the *wzy* mutation accumulates synthetic intermediates and depletes lipid carriers that are also needed for peptidoglycan synthesis (23, 24). This might explain the infrequent acquisition of the *wzy* mutant in this study. YA5 and YG2 form cell clusters due to the lack of peptidoglycan hydrolase activity required for daughter cell separation (25). CPSs have also been suggested to be involved in the bacterial aggregation phenotype (26), but *cps* derivatives from YG2 form cell clusters the same as YG2 (Fig. S8B), which refutes the relationship between CPSs and the cluster-forming phenotype in this case.

**TABLE 2:** Primers used in this study

Primer name	Sequence (5′→3′)
YA5_22620	TCGCGTTGGTAACGCAG
YA5_22670	GTGCGCATCCGAAGC
YA5_22670-2	AAACAGCGCAAGAAATGGTAG
YA5_22740	TTTAATTGAACTGCATTACGTACCC
YA5_22740-2	TCTGTTGTAGATGAAGTCGAGGG
YA5_22800	CCACACTCCGAATCTTCTCC
YA5_22800-2	TCGACAGTTCCGGTTGG
YA5_22880	TCAGCATGGCGGGG
YG2_06590	AATGAATGGTACTAACAACTCTCGG
YG2_06590-2	GGGAATTCGGACGCG
YG2_06630	CAAAGGTAACAGCTTTTTAGGGATG
YG2_16110	CCAGTACGATTTTCAAACGAGTC
YG2_16130	GGTACACTCGCTCGCTCC

### CPS analysis of the derivatives.

**FIG 2:**
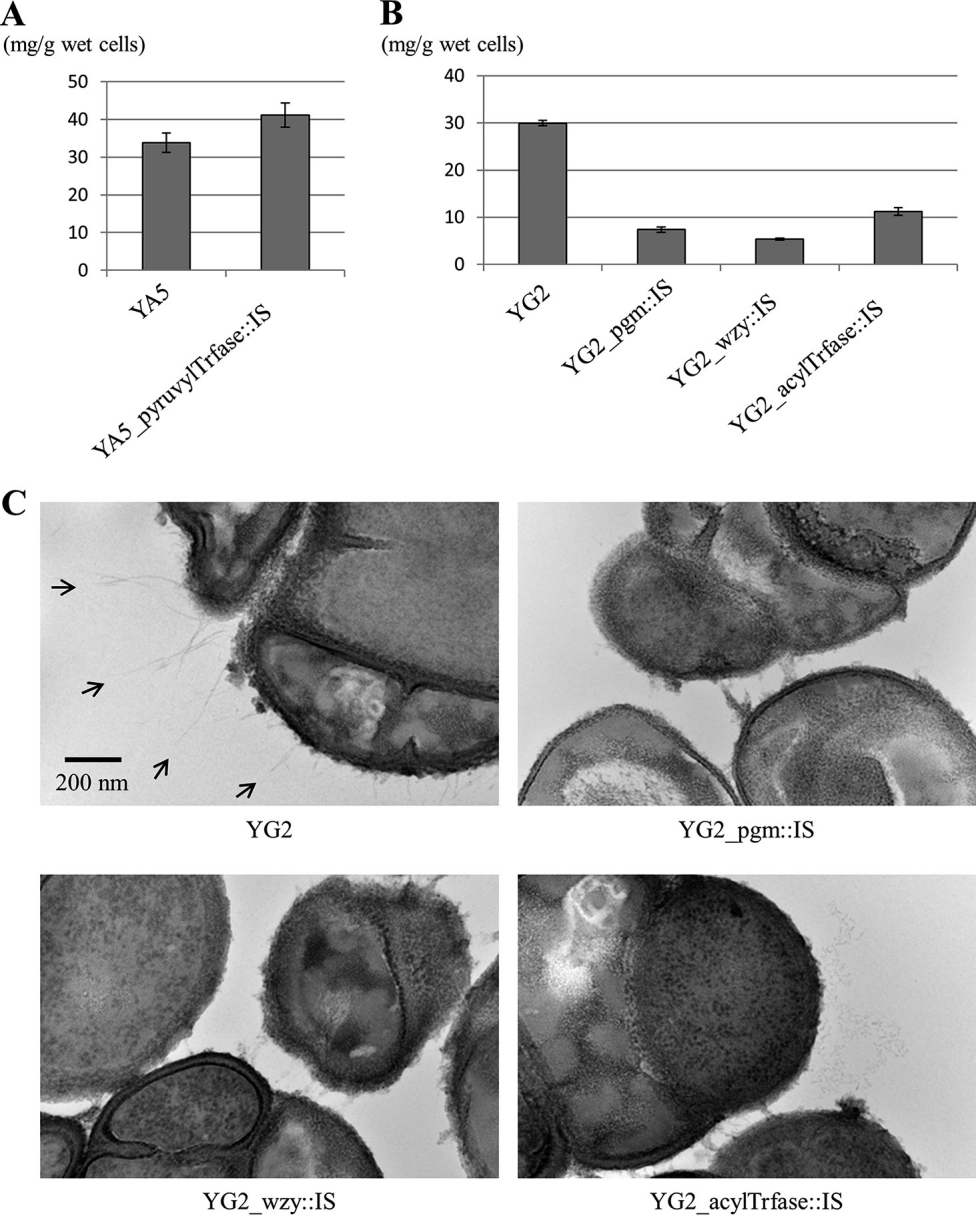
Analysis of cell surface polysaccharides in *T. halophilus*. (A) Quantification of polysaccharides in YA5 and its derivative. Data are expressed as the means, with error bars representing the standard deviations (SD) (*n* = 3). (B) Quantification of polysaccharides in YG2 and its derivatives. Data are expressed as the means, with error bars representing the SD (*n* = 3). (C) TEM images of YG2 and its derivatives. Arrows indicate the filamentous structures outside the cell walls of YG2.

To assess the CPS production of the *cps* derivatives, cell surface polysaccharides from YA5, YG2, and their derivatives were extracted and quantified by a phenol-sulfuric acid method (Fig. 2A and B). YA5_pyruvylTrfase::IS produced no fewer polysaccharides than the parental strain YA5. Therefore, YA5_pyruvylTrfase::IS is likely to produce adequate amounts of CPS, but it probably has a structural alteration. The three YG2 derivatives produced fewer glycopolymers than the parental strain, which shows that CPS production by the derivatives was severely damaged. α-Phosphoglucomutase prepares donor substrates for CPS synthesis, and the Wzy polymerase polymerizes CPS chains, which suggests that both genes are essential for CPS synthesis. Accordingly, we considered that CPS production in these derivatives is almost completely lost. The remaining reactants in these derivatives could be other types of cell surface polysaccharides such as WTA. On the other hand, acyltransferase is predicted to be involved in the decoration of CPSs and not indispensable for CPS synthesis, and YG2_acylTrfase::IS produced slightly more polysaccharides than YG2_pgm::IS and YG2_wzy::IS, which implies the possibility that CPS production by YG2_acylTrfase::IS is not completely lost. YG2 and its derivatives were visualized by transmission electron microscopy (TEM) (Fig. 2C). Filamentary structures (>400 nm for long ones) were observed outside the cell walls of YG2, but the three derivatives lacked such structures. These results demonstrated that the *cps* loci in *T. halophilus* are responsible for the production of CPSs, which were observed as filamentary capsule structures. Although we expected YG2_acylTrfase::IS to display a slight capsule, it seems that the amounts of CPSs in YG2_acylTrfase::IS were not large enough to be observed. Since some parts of the filaments are bleary, it is likely that the visible filaments are only part of the CPSs and that the CPSs, although invisible by TEM, possibly form a layer around cells. Particularly thick filaments might have been created by the aggregation of CPSs during TEM fixation/staining. The cell wall thickness of YG2_pgm::IS was decreased, probably because α-phosphoglucomutase contributes to the synthesis of not only CPS but also other polysaccharides such as WTA and LTA (27).

### *cps* derivatives have altered phage susceptibility.

**FIG 3:**
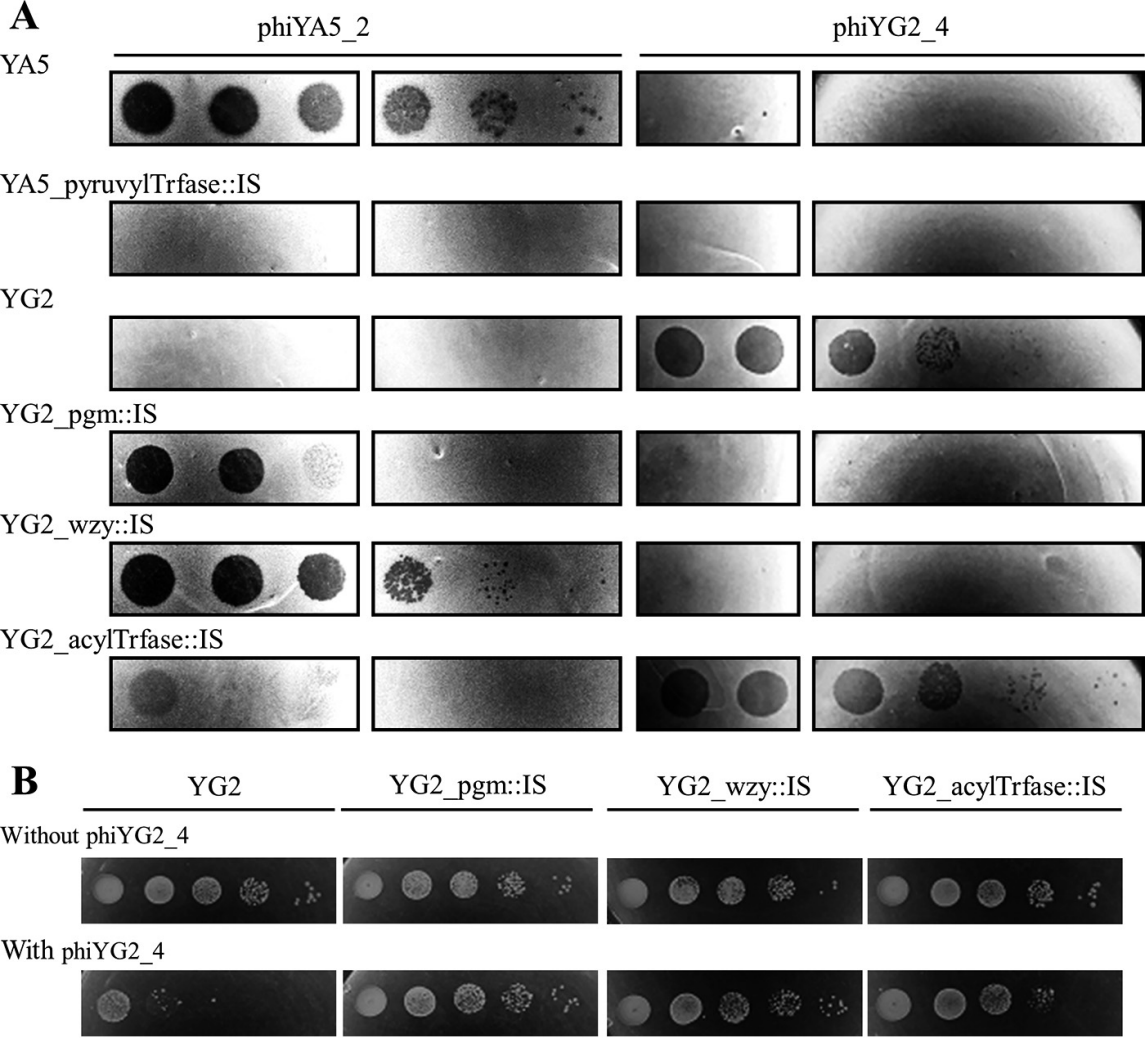
Phage susceptibility of YA5, YG2, and their derivatives. (A) phiYA5_2 and phiYG2_4 specimens were serially diluted 10-fold from left to right and spotted onto each host strain. (B) YG2 and its derivatives were grown with or without phiYG2_4. More than 10^6^ PFU of phiYG2_4 were plated prior to the spotting of cells. The cells were serially diluted 10-fold from left to right.

The susceptibility of the *cps* derivatives to phiYA5_2 and phiYG2_4 was investigated by plaque formation assays (Fig. 3A). As expected, YA5_pyruvylTrfase::IS was not infected by phiYA5_2. Surprisingly, the three *cps* derivatives from YG2 were susceptible to phiYA5_2, although the parent strain was not. YG2_pgm::IS and YG2_wzy::IS became resistant to phiYG2_4, but curiously, YG2_acylTrfase::IS was still susceptible to phiYG2_4, as was the parent strain, although it had been obtained as a phiYG2_4-resistant strain. To address this discrepancy, cultures of YG2 and its derivatives were diluted and spotted onto LA13 agar plates with or without phiYG2_4 (Fig. 3B). YG2_pgm::IS and YG2_wzy::IS were not affected by the presence of phiYG2_4. The growth of YG2_acylTrfase::IS was slightly inhibited, but the parent strain YG2 was further obstructed by phiYG2_4. Taken together, it was concluded that YG2_acylTrfase::IS acquired resistance to phiYG2_4 but was not insensitive.

### Bacteriophage adsorption to the *cps* derivatives.

**FIG 4:**
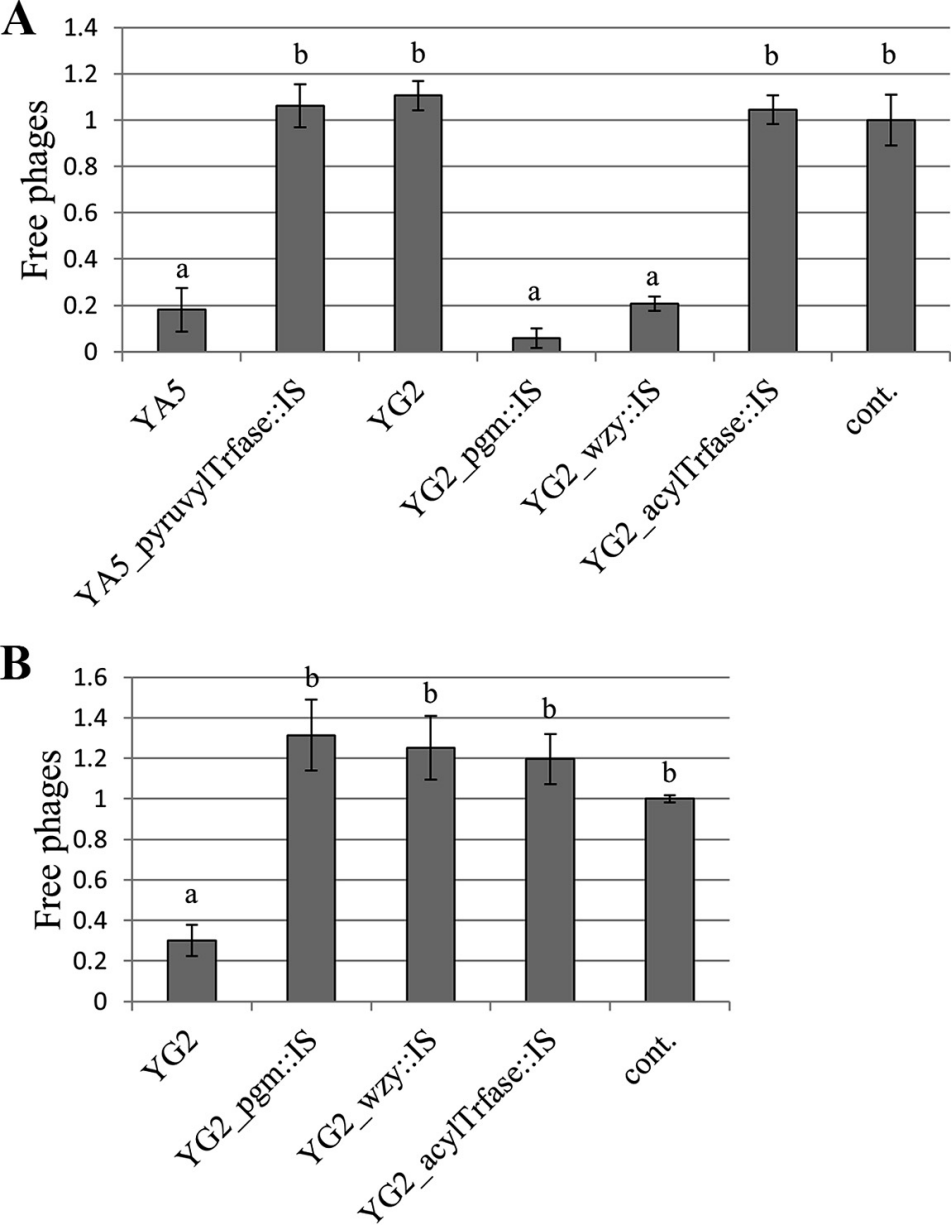
Phage adsorption to host cells. Free phage titers after centrifugation were calculated as a ratio of the control (cont.) (without cells). Data are expressed as the means, with error bars representing the SD (*n* = 3). Bars with different letters are significantly different by Tukey’s multiple-comparison test. (A) Adsorption by phiYA5_2. (B) Adsorption by phiYG2_4.

Bacterial CPSs are reported to act as barriers and/or receptors for phages (28, 29). Hence, we speculated that the adsorption of the phages to the *cps* derivatives was affected in some way that conferred phage resistance to the derivatives. We performed a phage adsorption assay with YA5, YG2, and their derivatives. The cells were mixed with the phages, and the bound phages were removed by centrifugation. Phage adsorption was measured from the free phage titers in the supernatant. YA5, YG2_pgm::IS, and YG2_wzy::IS were adsorbed by phiYA5_2 (Fig. 4A), consistent with the results of the plaque formation assay (Fig. 3A). These data verify the presence of a binding receptor for phiYA5_2 in both YA5 and YG2. The structural changes in CPSs that occurred in YA5_pyruvylTrfase::IS could have prevented adsorption by phiYA5_2, but the loss of CPSs of YG2 allowed phiYA5_2 to adsorb. These results indicated that CPSs are a barrier to rather than a receptor for phiYA5_2. It is likely that CPSs hide the other receptor molecules on the cell surface, but phiYA5_2 specifically breaks through the CPSs of YA5 and approaches the receptor (see below). The adsorption of phiYG2_4 to YG2 was also confirmed, but absorption to the three *cps* derivatives from YG2 was not. This is consistent with the resistance to phiYG2_4 (Fig. 3B). The fact that the CPSs of YG2 are required for adsorption by phiYG2_4 suggests that the CPSs of YG2 are specific receptors for phiYG2_4. The lack of structural decoration in CPSs of YG2_acylTrfase::IS perhaps made the adsorption by phiYG2_4 less efficient and conferred resistance against phiYG2_4 to YG2_acylTrfase::IS. It can also be guessed that phages could bind to hosts less efficiently when they were dispersed in liquid than when they were densely packed on an agar plate. The reason for the strong plaque formation of phiYG2_4 on YG2_acylTrfase::IS (Fig. 3A), despite the decreased adsorption efficiency, may be because the thinning of capsule facilitates phage infection, even if the phage targets capsule (30).

### phiYA5_2 possesses the CPS depolymerase.

**FIG 5:**
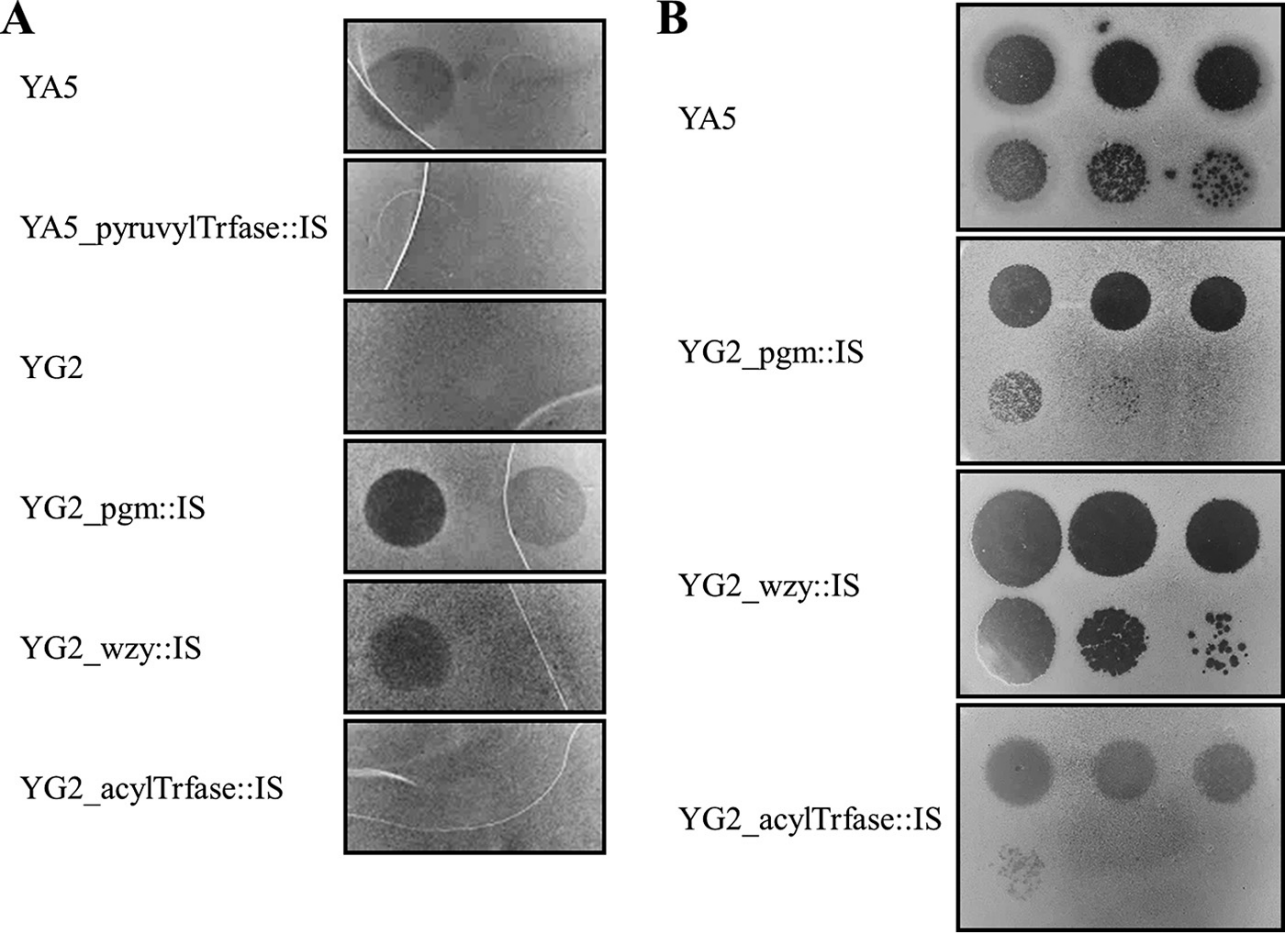
Cell-degrading enzyme activities of phiYA5_2. (A) UV-inactivated virions of phiYA5_2 were spotted onto each strain. The left spot contained 1.9 × 10^7^ PFU of virions (before UV irradiation), and the right spot contained 1/10 of that. (B) Plaques of phiYA5_2 were observed by shining light from behind the plates. The spotted phage specimens were serially diluted 10-fold from the top left to the bottom right.

As described above, it was estimated that CPSs prevented the adsorption of phiYA5_2 to host cells, but phiYA5_2 specifically overcame the CPSs of YA5. CPSs have been reported to act as physical barriers against phages, and phages are equipped with various virion-associated enzymes to penetrate CPSs, termed polysaccharide depolymerases (31). Virions of phiYA5_2 were spotted onto each strain after UV irradiation, which renders the virions noninfective but leaves the enzymatic activity of the viral proteins intact (32). The phage particles formed no plaques but created translucent zones on YA5, YG2_pgm::IS, and YG2_wzy::IS, which clearly shows that the virions of phiYA5_2 possess activities to degrade cell components of the host strains (Fig. 5A). This result is in good agreement with those of the adsorption experiment (Fig. 4A), suggesting that cell component degradation by virion-associated enzymes is a prerequisite for phage adsorption to receptors (or vice versa). Since these zones were formed not only on YA5 but also on CPS-deficient derivatives of YG2, we hypothesized that the zones were brought about by peptidoglycan degradation. Adsorption by multiple phages that cleave a pore in the bacterial cell wall causes instant cell lysis (33). Depolymerases can cause CPS degradation prior to infection and result in plaque-surrounding halos, which are the typical hallmarks of the presence of depolymerases (31). We carefully observed the plaques of phiYA5_2 and noticed turbid halos around the plaques on YA5 (Fig. 5B). Such halos were not formed around the plaques on YG2_pgm::IS, YG2_wzy::IS, and YG2_acylTrfase::IS, which suggests that phiYA5_2 possesses the depolymerase digesting the CPSs of YA5. The fact that YG2 cells were not lysed but the *cps* derivatives of YG2 were lysed by UV-inactivated phiYA5_2 supports that CPSs are a barrier for peptidoglycan hydrolase to contact cell walls. Taken together, we consider that the cell lysis of YA5 by inactivated phiYA5_2 was synergistically caused by CPS depolymerase and peptidoglycan hydrolase, while the cell lysis of YG2_pgm::IS and YG2_wzy::IS was caused by peptidoglycan hydrolase alone. YG2_pgm::IS was more susceptible to the UV-inactivated virions of phiYA5_2 than YA5 and YG2_wzy::IS, possibly because the disruption of α-phosphoglucomutase affects the other cell wall components and makes YG2_pgm::IS more vulnerable to peptidoglycan hydrolase. Given the possibility that YA5_pyruvylTrfase::IS has structural alterations in CPSs, the specific depolymerase of phiYA5_2 may not be able to recognize the altered CPS structure of YA5_pyruvylTrfase::IS, which probably explains why phiYA5_2 cannot approach the cell surface of YA5_pyruvylTrfase::IS and cannot infect it. To predict the gene encoding the depolymerase from phiYA5_2, we compared the genome of phiYA5_2 to that of phiWJ7, which belongs to the same genus as phiYA5_2. Overall, the structures of the two phage genomes were quite similar, but the ORFs encoding phage tail fiber proteins were remarkably different, which suggests that tail fibers might be responsible for host specificity (Fig. S9). Considering that most depolymerases are encoded by the ORF of the tail spike or tail fiber (34), the tail fiber of phiYA5_2 is likely to possess depolymerase activity, degrading the CPSs of YA5. We previously indicated that the tail spike of phiWJ7 is homologous to the peptidoglycan-degrading enzyme of staphylococcal phages (6), and phiYA5_2 also possessed a similar tail spike, suggesting that the tail spike of phiYA5_2 is responsible for peptidoglycan digestion.

## DISCUSSION

### Identification of CPS.

In this study, we identified CPS as the factor determining phage susceptibility in *T. halophilus*. This information could help establish effective strategies to avoid fermentation failures caused by phage-induced lysis of starter strains, e.g., by preparing a mixture of strains with different phage susceptibilities as a starter culture.

We recently identified WTA as an indispensable irreversible binding receptor for the *T. halophilus* phage phiWJ7, but another cell wall component was estimated to be involved in the first reversible attachment step (6). Moreover, the genetic diversity of WTA synthesis genes was not high enough to explain the observed narrow host ranges of tetragenococcal phages. Therefore, we conducted this study and concluded that the *cps* loci contributing to CPS synthesis were the genetic determinants of phage susceptibility in *T. halophilus* and that the variability in the *cps* loci is at least partially responsible for the narrow host ranges of tetragenococcal phages. The structural diversity of CPSs from Gram-negative bacteria often limits the host range of phages, but this study is meaningful in that it suggests a similar mechanism in Gram-positive bacteria.

Bacterial CPSs exhibit great diversity in sugar composition, linkage, and branching. Determination of the detailed CPS structures is particularly challenging as the diversity of CPS components and branching complicate structural analyses. Very recently, EPS structures from *T. halophilus* were analyzed, and they contained various components such as glucose, galactose, mannose, arabinose, xylose, fucose, and glucuronic acid (35, 36). Structural analyses of CPSs in *T. halophilus* are expected to be conducted in other studies.

Capsules are produced by many bacterial species, including both Gram-positive and Gram-negative bacteria, but their morphological characteristics are diverse. For instance, some strains of Staphylococcus aureus produce a thick capsule that can be visualized by light microscopy with India ink staining (37). On the other hand, a capsule from E. faecium cannot be seen without immunogold labeling, even by TEM (38). The capsule from *T. halophilus* was not detectable by light microscopy (data not shown), but a part of it could be observed as filamentary structures by TEM (Fig. 2C). Similar filamentary structures from *T. halophilus* were previously observed by Ueki et al., although their identification has not been obtained (39).

### CPS as a receptor/barrier.

CPSs are known to act as a protective barrier against stress factors such as ethanol, low pH, lysozyme, and antibiotics (40, 41). In this study, it was shown that CPSs can protect *T. halophilus* against phage infections, and other roles of CPSs in *T. halophilus* will be revealed in the future.

Since phiYG2_4 could not bind to *cps* derivatives of YG2, we considered that CPSs are the binding receptors for phiYG2_4 (Fig. 4B). Several types of cell surface polysaccharides of Gram-positive bacteria, in addition to peptidoglycan, WTA, and LTA, have been reported to be receptors for phage adsorption. For instance, CWPSs, the so-called “pellicles” of L. lactis, are targeted by phages and mainly define phage susceptibility in this species (42). EPSs of Streptococcus thermophilus are the receptors for streptococcal phage CHPC926 (43). Enterococcal phage ΦNPV1 utilizes a complex polysaccharide called “enterococcal polysaccharide antigen” (EPA) of Enterococcus faecalis (44). Thus, various glycopolymers can serve as phage binding receptors, and CPSs have also been reported to be receptors in many Gram-negative bacteria such as Escherichia coli, Campylobacter jejuni, and Salmonella enterica (28, 45). However, only Clostridium perfringens phage CPS1 is known to date as a phage infecting Gram-positive bacteria using CPS as a receptor (46). To our knowledge, phages infecting lactic acid bacteria have not yet been reported to target CPSs as receptors. To irrefutably prove that CPS is the binding receptor for phiYG2_4, we preincubated phiYG2_4 with CPSs extracted from YG2 prior to infection, but a decrease in PFU was not observed (data not shown). Ho et al. obtained a similar result when ΦNPV1 was preincubated with EPA, and they speculated that EPA is not the only requirement for ΦNPV1 adsorption or that the availability of EPA for ΦNPV1 differs in whole cells versus EPA extracts (44). In any case, further research is necessary to elucidate the detailed mechanism by which CPSs in *T. halophilus* are involved in phage adsorption.

It is also known that CPSs act as a barrier to sterically inhibit phage adsorption (29). To break through this barrier, phages utilize CPSs as receptors and/or degrade CPSs by CPS depolymerases (30). The depolymerases digest CPSs, thereby drilling a tunnel through the capsules and enabling phage particles to contact the receptors under the capsules. The high level of diversity of CPS structures can contribute to the narrow host spectrum, as phage-carried CPS depolymerases recognize specific CPS structures. A considerable number of putative depolymerases encoded by phages infecting Gram-negative pathogens such as Klebsiella pneumoniae, Pseudomonas aeruginosa, and Acinetobacter baumannii have been identified, but only scarce information is available about depolymerases from phages infecting Gram-positive bacteria (47). Hyaluronidases that act on hyaluronan capsules from Streptococcus pyogenes and Streptococcus equi and γ-glutamyl hydrolases that act on poly-γ-glutamate capsules from Bacillus subtilis are known (48–50). We predicted that the tail fiber gene of phiYA5_2 encodes the depolymerase (see Fig. S9 in the supplemental material), and the precise identification of phage-encoded depolymerases that digest strain-specific CPSs of Gram-positive bacteria, including *T. halophilus*, will be an interesting topic for future studies.

### Insertion sequences.

ISs played important roles in this study. We previously reported the very frequent transposition of three IS*4* family ISs, IS*Teha3*, IS*Teha4*, and IS*Teha5*, in *T. halophilus* (6, 22). Here, we discovered two additional active ISs, named IS*Teha7* and IS*Teha8*, for transposition into the α-phosphoglucomutase gene of YG2 (Fig. S3 and S6). Based on the amino acid sequence relatedness of the transposases, we entered IS*Teha7* as a novel IS*5* family IS*5* subgroup member and IS*Teha8* as a novel IS*4* family IS*Pepr1* subgroup member in the ISfinder database. Four of the five ISs identified as being active in *T. halophilus* to date are IS*4* family members, and IS*Teha7* is the only IS*5* family member. The three previously described ISs are distributed in the genomes of all six *T. halophilus* strains whose complete genomes were determined. However, one strain among the six does not possess IS*Teha7*, and two strains among the six do not possess IS*Teha8*, suggesting that these ISs are minor mutagens compared to the other three ISs. ISs are utilized in transposon mutagenesis systems for the characterization of gene functions (51). They are also used for the identification of phage receptors (52). Usually, transposon mutagenesis is accomplished by inserting ISs with a drug resistance marker gene from an extrinsic plasmid into the host chromosome, but we could execute this study with intrinsic ISs without marker genes due to the surprisingly high transposition frequency of ISs in *T. halophilus*. We will continue to pursue research to understand the basic features of ISs in *T. halophilus* and develop their utility.

## MATERIALS AND METHODS

### Bacterial strains, bacteriophages, media, and culture conditions.

The bacterial strains and phages mainly used in this study are summarized in Table 1. Derivatives of YA5 and YG2 were obtained as described below and are listed in Table S1 in the supplemental material. *T. halophilus* strains were cultured in MRS-10 or LA13 medium (53). MRS-10 medium is lactobacillus MRS broth (Difco, Detroit, MI) supplemented with 10% NaCl. Liquid media were statically incubated, and agar plates were incubated under anaerobic conditions using an AnaeroPack system (Mitsubishi Gas Chemical, Tokyo, Japan) at 30°C. Cell morphology was observed using a BX53 optical microscope (Olympus, Tokyo, Japan).

### Manipulation of bacteriophages and generation of phage-resistant mutants.

Uchida and Kanbe previously developed a method for the manipulation of tetragenococcal phages (4), and we modified this method as described previously (6). Briefly, instead of using the general soft agar overlay technique, the bacterial suspension was diluted with saline and spread onto LA13 agar plates by tilting the plates to form plaques. To obtain spontaneous phage-resistant mutants, the parental strain was incubated with >10^7^ PFU of phages on LA13 plates, and the surviving colonies were selected. To observe the enzymatic activity of the virions, phage particles were UV irradiated using a germicidal lamp (GL-15; Panasonic, Kadoma, Japan) at a distance of 50 mm for 10 min.

### DNA preparation and genome sequencing.

Genomic DNA from *T. halophilus* was isolated using the DNeasy PowerSoil Pro kit (Qiagen, Hilden, Germany) and QIAcube, an automated system (Qiagen). Genomic DNA from bacteriophage phiYA5_2 was isolated using a phage DNA isolation kit (Norgen Biotek, Thorold, Canada). The quantity and purity of the DNA were assessed with a Qubit dsDNA BR (double-stranded DNA broad-range) assay kit (Thermo Fisher Scientific, Waltham, MA) and a NanoDrop 1000 spectrophotometer (Thermo Fisher Scientific). An Illumina (San Diego, CA) Nextera DNA Flex library prep kit was used to prepare the genomic DNA library. Whole-genome sequencing was conducted using the Illumina MiSeq sequencing platform with a paired-end sequencing strategy (2× 300 bp). Adapter sequences and low-quality regions in the Illumina reads were trimmed using Trim Galore! v.0.6.4 with default parameters (https://www.bioinformatics.babraham.ac.uk/projects/trim_galore/). The *de novo* assembly of phage genomes was conducted as previously described (6). Briefly, read data were mapped onto the host genome to remove the contaminated host genome sequence, and unmapped read pairs were used for *de novo* assembly with SPAdes v.3.13.0 (54). The DDBJ Fast Annotation and Submission Tool was used for gene detection and genome annotation of the draft genome assemblies, with default settings (55).

### Genome mapping analysis and search for mutations in the derivatives.

To detect the mutation sites of YA5R1, YA5R1R1, and YG2R1, the genome data for *T. halophilus* YA5, YA5R1, and YG2 were used as the reference sequences for our genome mapping analysis. The genome sequences of YA5 (DRA accession number DRR424329) and YG2 (accession number DRR220997) were previously published (56). The Illumina sequence reads of the derivatives were mapped to the reference sequence of the parental strain using BWA with default parameters (57). Single nucleotide polymorphisms and indels were detected using the Genome Analysis Toolkit (GATK) (58, 59). For the detection of transposon insertion sites, breseq v.0.31.0 was used with default parameters (60). To find the IS transposition in the additionally acquired derivatives YA5R2 to YA5R17 and YG2R2 to YG2R49, the primer sets YA5_22620 and YA5_22670, YA5_22670-2 and YA5_22740, YA5_22740-2 and YA5_22800, YA5_22800-2 and YA5_22880, YA5_22670-2 and YG2_06590, YG2_06590-2 and YG2_06630, and YG2_16110 and YG2_16130 were used to amplify the DNA regions covering the CPS synthesis genes (Table 2 and Fig. 1). The PCR products amplified with KOD FX Neo DNA polymerase (Toyobo, Osaka, Japan) were purified using the Wizard SV gel and PCR cleanup system (Promega, Madison, WI), and the resulting DNA fragment was analyzed by a commercial DNA sequencing service (Fasmac, Atsugi, Japan/Eurofins Genomics, Tokyo, Japan).

### CPS extraction and analysis.

CPSs were roughly quantified according to a method described previously by Ha et al. (46). Briefly, cells were grown to stationary phase in MRS-10 medium and harvested by centrifugation (8,000 × *g* for 5 min at 4°C). The pellets (0.2 g) were washed with sodium phosphate buffer (50 mM, pH 7.0) and suspended in 2 mL of sodium phosphate buffer containing 70 mg of lysozyme. After incubation at 37°C for 24 h, the supernatant was recovered by centrifugation and treated with DNase, RNase, and proteinase K. The supernatant was adjusted to a concentration of 30% (vol/vol) ethanol and cooled at 4°C for 2 h, and the precipitate was removed by centrifugation. Next, the supernatant was adjusted to a concentration of 80% ethanol and precipitated. CPSs were collected by centrifugation and lyophilized. For quantification analysis of CPSs, a modified phenol-sulfuric acid method was employed. The dried pellet was resuspended in 600 μL of water, and 300 μL of 5% phenol was added. Next, 1.5 mL of 95% sulfuric acid was added, and the color of the mixture was allowed to develop for 10 min at room temperature. Finally, the intensity of the color was measured at 490 nm with a spectrophotometer. The concentration of the extracted CPSs was calculated based on the color intensity of the glucose standard. For sugar composition analysis, hydrolysates of CPSs (2 M HCl at 100°C for 3 h) were analyzed with an LC-20 reducing sugar analysis system (Shimadzu, Kyoto, Japan). For imaging, cells cultured in MRS-10 medium were collected by centrifugation, fixed with 2% glutaraldehyde in 0.1 M cacodylate buffer (pH 7.0), and stained with 2% osmium tetroxide containing 0.8% sucrose. The cells were then stepwise dehydrated in ethanol solutions of increasing concentrations and embedded in Epon 812. Ultrathin sections were prepared and stained with 2% uranyl acetate and lead. TEM observation was carried out using an H-7600 microscope (Hitachi Ltd., Tokyo, Japan) at Hanaichi UltraStructure Research Institute (Okazaki, Japan).

### Bacteriophage adsorption assay.

Phage adsorption to cells was measured according to a method described previously by Baptista et al. (61). Cells were grown in LA13 liquid medium to an optical density (OD) of 0.5 and sterilized by heating at 60°C for 30 min. Phages were mixed with cells and incubated at 30°C for 2 h. Control mixtures without cells were used to confirm the phage input in the experiment. After incubation, the mixture was centrifuged (6,000 × *g* for 5 min at 4°C), and the supernatant was assayed for plaques using YA5 for phiYA5 and phiYA5_2 and YG2 for phiYG2_4 as the indicator strains. Unbound free phages in the supernatants after centrifugation were enumerated.

### Statistical analysis.

The data were analyzed using R software (version 3.6.0) (www.r-project.org). Comparisons of two groups were performed using an unpaired *t* test. Data involving more than two groups were assessed by one-way analysis of variance (ANOVA) followed by Tukey’s *post hoc* multiple-comparison test. Statistical significance was considered at a *P* value of <0.05.

### Data availability.

BLAST was used for the DNA and amino acid sequence analyses. Illumina sequence reads of YA5R1, YA5R1R1, and YG2R1 were deposited in the DDBJ Sequence Read Archive. The DRA accession numbers for YA5R1, YA5R1R1, YG2R1, and phiYA5_2 are DRR424330, DRR424331, DRR424332, and DRR424333, respectively. The BioProject accession numbers are PRJDB9642 for DRA accession numbers DRR424330, DRR424331, and DRR424332 and PRJDB12096 for DRA accession number DRR424333. Other sequence data were deposited in the DDBJ database with accession numbers LC720293 to LC720358.
