# Supplementary material for: Identification of Capsular Polysaccharide Synthesis Loci Determining Bacteriophage Susceptibility in Tetragenococcus halophilus
Source: Microbiol Spectr. 2023 May 8;11(3):e00385-23. doi: 10.1128/spectrum.00385-23 (PMC10269466; doi:10.1128/spectrum.00385-23)
Supplement: Supplemental file 1 — Supplemental material. Download spectrum.00385-23-s0001.pdf, PDF file, 0.8 MB [file spectrum.00385-23-s0001.pdf]

Table S1. Bacterial derivatives generated in this study

	Description	genotype
YA5R1	PhiYA5-resistant derivative from YA5.	
YA5R1R1	PhiYA5_2-resistant derivative from YA5R1.	YA5_22780::IS*Teha3*
YA5R2 (YA5_pyruvylTrfase::IS)	PhiYA5_2-resistant derivative from YA5.	YA5_22780::IS*Teha4*
YA5R3	PhiYA5_2-resistant derivative from YA5.	YA5_22780::IS*Teha3*
YA5R4	PhiYA5_2-resistant derivative from YA5.	YA5_22780::IS*Teha3*
YA5R5	PhiYA5_2-resistant derivative from YA5.	YA5_22780::IS*Teha3*
YA5R6	PhiYA5_2-resistant derivative from YA5.	YA5_22780::IS*Teha4*
YA5R7	PhiYA5_2-resistant derivative from YA5.	YA5_22780::IS*Teha4*
YA5R8	PhiYA5_2-resistant derivative from YA5.	YA5_22780::IS*Teha5*
YA5R9	PhiYA5_2-resistant derivative from YA5.	YA5_22780::IS*Teha5*
YA5R10	PhiYA5_2-resistant derivative from YA5.	YA5_22780::IS*Teha4*
YA5R11	PhiYA5_2-resistant derivative from YA5.	YA5_22780::IS*Teha3*
YA5R12	PhiYA5_2-resistant derivative from YA5.	YA5_22780::IS*Teha3*
YA5R13	PhiYA5_2-resistant derivative from YA5.	YA5_22780::IS*Teha3*
YA5R14	PhiYA5_2-resistant derivative from YA5.	YA5_22780::IS*Teha4*
YA5R15	PhiYA5_2-resistant derivative from YA5.	YA5_22780::IS*Teha3*
YA5R16	PhiYA5_2-resistant derivative from YA5.	YA5_22780::IS*Teha4*
YA5R17	PhiYA5_2-resistant derivative from YA5.	YA5_22780::IS*Teha3*

YG2R1 (YG2_pgm::IS)	PhiYG2_4-resistant derivative from YG2.	YG2_16120::IS*Teha3*
YG2R2 (YG2_wzy::IS)	PhiYG2_4-resistant derivative from YG2.	YG2_06590::IS*Teha3*
YG2R3	PhiYG2_4-resistant derivative from YG2.	YG2_16120::IS*Teha3*
YG2R4	PhiYG2_4-resistant derivative from YG2.	YG2_16120::IS*Teha4*
YG2R5	PhiYG2_4-resistant derivative from YG2.	YG2_16120::IS*Teha3*
YG2R6	PhiYG2_4-resistant derivative from YG2.	YG2_16120::IS*Teha4*
YG2R7	PhiYG2_4-resistant derivative from YG2.	YG2_16120::IS*Teha4*
YG2R8	PhiYG2_4-resistant derivative from YG2.	YG2_16120::IS*Teha3*
YG2R9	PhiYG2_4-resistant derivative from YG2.	YG2_16120::IS*Teha4*
YG2R10	PhiYG2_4-resistant derivative from YG2.	YG2_16120::IS*Teha4*
YG2R11	PhiYG2_4-resistant derivative from YG2.	YG2_16120::IS*Teha4*
YG2R12	PhiYG2_4-resistant derivative from YG2.	YG2_16120::IS*Teha4*
YG2R13	PhiYG2_4-resistant derivative from YG2.	YG2_16120::IS*Teha4*
YG2R14	PhiYG2_4-resistant derivative from YG2.	YG2_16120::IS*Teha4*
YG2R15	PhiYG2_4-resistant derivative from YG2.	YG2_16120::IS*Teha4*
YG2R16	PhiYG2_4-resistant derivative from YG2.	YG2_16120::IS*Teha4*
YG2R17	PhiYG2_4-resistant derivative from YG2.	YG2_16120::IS*Teha4*
YG2R18	PhiYG2_4-resistant derivative from YG2.	YG2_16120::IS*Teha4*
YG2R19	PhiYG2_4-resistant derivative from YG2.	YG2_16120::IS*Teha4*
YG2R20	PhiYG2_4-resistant derivative from YG2.	YG2_16120::IS*Teha3*
YG2R21	PhiYG2_4-resistant derivative from YG2.	YG2_16120::IS*Teha4*
YG2R22	PhiYG2_4-resistant derivative from YG2.	YG2_16120::IS*Teha4*
YG2R23	PhiYG2_4-resistant derivative from YG2.	YG2_16120::IS*Teha4*
YG2R24	PhiYG2_4-resistant derivative from YG2.	YG2_16120::IS*Teha4*
YG2R25	PhiYG2_4-resistant derivative from YG2.	YG2_16120::IS*Teha4*
YG2R26	PhiYG2_4-resistant derivative from YG2.	YG2_16120::IS*Teha4*
YG2R27	PhiYG2_4-resistant derivative from YG2.	YG2_16120::IS*Teha4*
YG2R28	PhiYG2_4-resistant derivative from YG2.	YG2_16120::IS*Teha4*
YG2R29	PhiYG2_4-resistant derivative from YG2.	YG2_16120::IS*Teha4*
YG2R30	PhiYG2_4-resistant derivative from YG2.	YG2_16120::IS*Teha4*
YG2R31	PhiYG2_4-resistant derivative from YG2.	YG2_16120::IS*Teha4*
YG2R32	PhiYG2_4-resistant derivative from YG2.	YG2_16120::IS*Teha3*
YG2R33	PhiYG2_4-resistant derivative from YG2.	YG2_16120::IS*Teha8*
YG2R34	PhiYG2_4-resistant derivative from YG2.	YG2_16120::IS*Teha4*
YG2R35	PhiYG2_4-resistant derivative from YG2.	YG2_16120::IS*Teha3*
YG2R36	PhiYG2_4-resistant derivative from YG2.	YG2_16120::IS*Teha4*
YG2R37	PhiYG2_4-resistant derivative from YG2.	YG2_16120::IS*Teha4*
YG2R38	PhiYG2_4-resistant derivative from YG2.	YG2_16120::IS*Teha4*
YG2R39	PhiYG2_4-resistant derivative from YG2.	YG2_16120::IS*Teha4*
YG2R40	PhiYG2_4-resistant derivative from YG2.	YG2_16120::IS*Teha3*
YG2R41	PhiYG2_4-resistant derivative from YG2.	YG2_16120::IS*Teha7*
YG2R42	PhiYG2_4-resistant derivative from YG2.	YG2_16120::IS*Teha4*
YG2R43	PhiYG2_4-resistant derivative from YG2.	YG2_16120::IS*Teha4*
YG2R44	PhiYG2_4-resistant derivative from YG2.	YG2_06600::IS*Teha3*
YG2R45	PhiYG2_4-resistant derivative from YG2.	YG2_06600::IS*Teha4*
YG2R46	PhiYG2_4-resistant derivative from YG2.	YG2_06600::IS*Teha4*
YG2R47	PhiYG2_4-resistant derivative from YG2.	YG2_06600::IS*Teha4*
YG2R48 (YG2_acylTrfase::IS)	PhiYG2_4-resistant derivative from YG2.	YG2_06600::IS*Teha4*
YG2R49	PhiYG2_4-resistant derivative from YG2.	YG2_06600::IS*Teha4*

Table S2. DNA sequences of the insertion sites and their flanking regions of ISs in each derivative.

IS transposed in each derivative	Flanking sequence at 5' terminal	Insertion site (Duplicated target sequence)	Flanking sequence at 3' terminal
IS*Teha3* in YA5R1R1	AAATGTATTG	TAATAAA	CATACAGATC
IS*Teha4* in YA5R2	ATTCATTCTA	TTAATTA	TTAAAATTTG
IS*Teha3* in YA5R3	TGGAGGTAAT	TTATAAT	GATAACCAAG
IS*Teha3* in YA5R4	TTTATAATGA	TAACCAA	GATAAAAAAA
IS*Teha3* in YA5R5	AAATGTATTG	TAATAAA	CATACAGATC
IS*Teha4* in YA5R6	ATTCATTCTA	TTAATTA	TTAAAATTTG
IS*Teha4* in YA5R7	CAAATTTTAA	TAATTAA	TAGAATGAAT
IS*Teha5* in YA5R8	AATATTTTTT	TCTTCATTT	ATTCAATGGA
IS*Teha5* in YA5R9	TATAACCTTT	AGAATGTGC	GTATTTTATA
IS*Teha4* in YA5R10	CAAATTTTAA	TAATTAA	TAGAATGAAT
IS*Teha3* in YA5R11	GCTAAACAAT	TTATAAA	ATACGCACAT
IS*Teha3* in YA5R12	GCTTCTAATA	TAAATGA	ATCTGCTGAG
IS*Teha3* in YA5R13	GATCTGTATG	TTTATTA	CAATACATTT
IS*Teha4* in YA5R14	CTGTTAGGCA	TTACTAGT	CTATTTCGTC
IS*Teha3* in YA5R15	TGGGCTAAAT	ATATAA	TCTGCATGTT
IS*Teha4* in YA5R16	ACCTTTATAG	TCCATAA	GATATTGATT
IS*Teha3* in YA5R17	ACTCGGATAT	TTTTAT	CATTAAAGAG

IS*Teha3* in YG2R1	TGTTTTTAAG	TAA	CGCACTGCAA
IS*Teha3* in YG2R2	GTTTTTTTAT	CTACTTA	GTTATTTTAA
IS*Teha4* in YG2R3	ATGGAATATT	ATTATAA	GCTAATGTTT
IS*Teha4* in YG2R4	GGAGCTTTAC	TTATTCA	TTATATTTTA
IS*Teha3* in YG2R5	AAAAGGCATC	TTCTAA	CTCCTCTTGC
IS*Teha4* in YG2R6	GCGGAAAAAA	TTAAAGAA	TTTGAAGCAG
IS*Teha4* in YG2R7	AAACATTAGC	TTA	TAATAATATT
IS*Teha3* in YG2R8	TTCCTTTAAC	TCATCTA	ATTGTGTTTT
IS*Teha4* in YG2R9	TAAAATATAA	TGAATAA	GTAAAGCTCC
IS*Teha4* in YG2R10	ATCCTATGTA	TTTGAAA	GTTTAAGACC
IS*Teha4* in YG2R11	TAATTTTAGG	TTCAGTA	CCTGAAGGAC
IS*Teha4* in YG2R12	GCGGAAAAAA	TTAAAGAA	TTTGAAGCAG
IS*Teha4* in YG2R13	TAAAGCTTTC	TCACCTAA	CATTTTGCCC
IS*Teha4* in YG2R14	CGAATTAAAG	TTGTTAC	CATATCCGCA
IS*Teha4* in YG2R15	TAAAATATAA	TGAATAA	GTAAAGCTCC
IS*Teha4* in YG2R16	GGATGGAATA	TTATTATAA	GCTAATGTTT
IS*Teha4* in YG2R17	AATTTAAATC	CTGTTAA	AACATTAACC
IS*Teha4* in YG2R18	TAAAATATAA	TGAATAA	GTAAAGCTCC
IS*Teha4* in YG2R19	TAAAATATAA	TGAATAA	GTAAAGCTCC
IS*Teha3* in YG2R20	TCTGTCGCAA	TTAATAA	ATCAGCATTT
IS*Teha4* in YG2R21	CTGCTTCAAA	TTCTTTAA	TTTTTTCCGC
IS*Teha4* in YG2R22	GTCCTTCAGG	TACTGAA	CCTAAAATTA
IS*Teha4* in YG2R23	CTGCTTCAAA	TTCTTTAA	TTTTTTCCGC
IS*Teha4* in YG2R24	GCGGAAAAAA	TTAAAGAA	TTTGAAGCAG
IS*Teha4* in YG2R25	TAAAATATAA	TGAATAA	GTAAAGCTCC
IS*Teha4* in YG2R26	TATCATCTTC	TAAGGTA	TATTTCAAAA
IS*Teha4* in YG2R27	TTCTAAAATA	TAATGAA	TAAGTAAAGC
IS*Teha4* in YG2R28	ATGGAATATT	ATTATAA	GCTAATGTTT
IS*Teha4* in YG2R29	ATGGAATATT	ATTATAA	GCTAATGTTT
IS*Teha4* in YG2R30	CTAAAATATA	ATGAATAA	GTAAAGCTCC
IS*Teha4* in YG2R31	AAACATTAGC	TTATAATAA	TATTCCATCC
IS*Teha3* in YG2R32	ACTTCACGAA	TTAAAGT	TGTTACCATA
IS*Teha8* in YG2R33	GGAGCTTTAC	TTATTCA	TTATATTTTA
IS*Teha4* in YG2R34	CTAAAATATA	ATGAATAA	GTAAAGCTCC
IS*Teha3* in YG2R35	AAGGAAAAAC	ATTGTAT	GATGCCTTAC
IS*Teha4* in YG2R36	GGAGCTTTAC	TTATTCA	TTATATTTTA
IS*Teha4* in YG2R37	GGAGCTTTAC	TTATTCA	TTATATTTTA
IS*Teha4* in YG2R38	GATGGAATAT	TATTATAA	GCTAATGTTT
IS*Teha4* in YG2R39	GCGGAAAAAA	TTAAAGAA	TTTGAAGCAG
IS*Teha3* in YG2R40	GGATGGAATA	TTATTATAA	GCTAATGTTT
IS*Teha7* in YG2R41	AATTAAATTC	TATATA	GGTGTGAAAG
IS*Teha4* in YG2R42	CTGCTTCAAA	TTCTTTAA	TTTTTTCCGC
IS*Teha4* in YG2R43	TAAAATATAA	TGAATAA	GTAAAGCTCC
IS*Teha3* in YG2R44	GCACCTTTAT	TTACCA	TATGCCCATC
IS*Teha4* in YG2R45	TAACTTTTAC	TTTATAA	TTTATTAAAA
IS*Teha4* in YG2R46	TAACTTTTAC	TTTATAA	TTTATTAAAA
IS*Teha4* in YG2R47	TAACTTTTAC	TTTATAA	TTTATTAAAA
IS*Teha4* in YG2R48	TTTTAATAAA	TTATAAA	GTAAAAGTTA
IS*Teha4* in YG2R49	TAACTTTTAC	TTTATAA	TTTATTAAAA

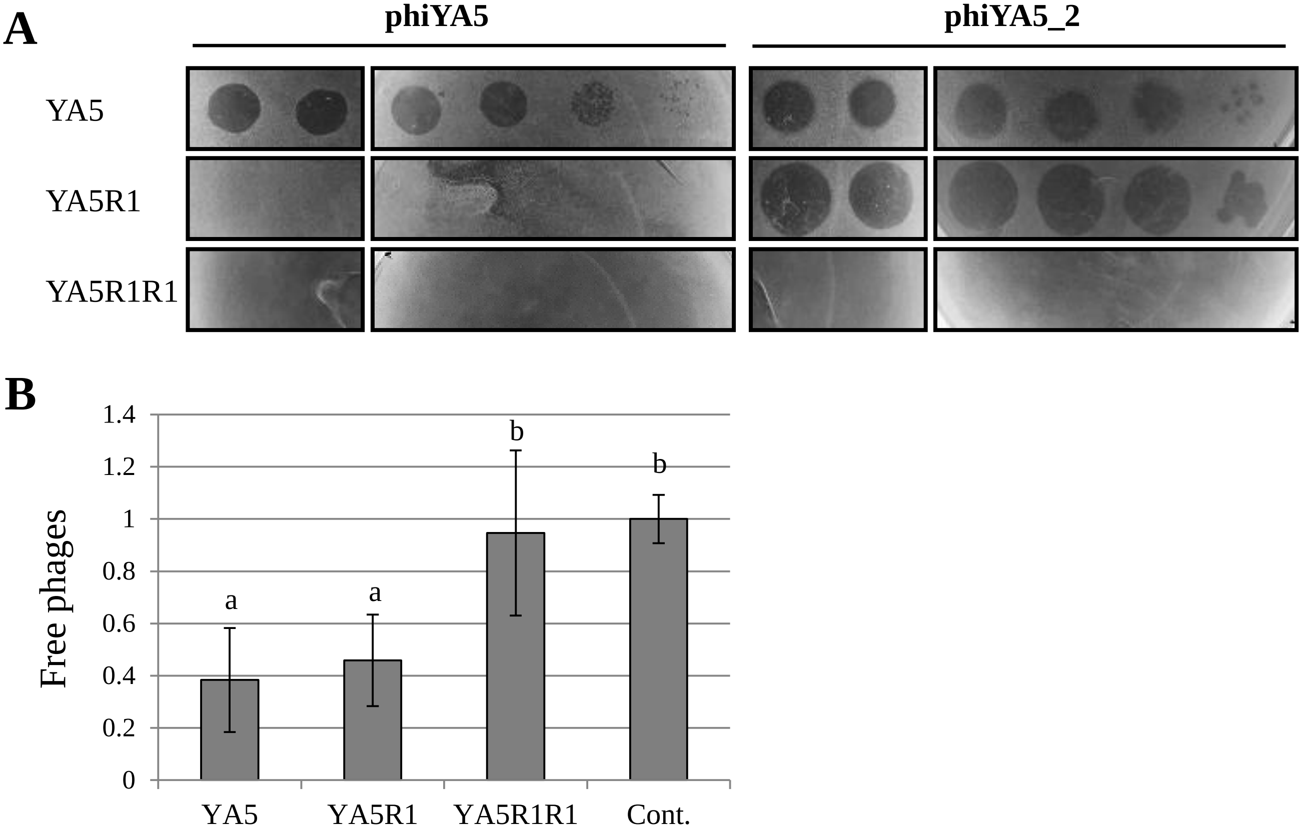

Figure S1: Phage susceptibility and adsorption of YA5, YA5R1, and YA5R1R1. (A) PhiYA5 and phiYA5_2 specimens were serially diluted 10-fold from left to right and spotted on each host strain. (B) Adsorption by phiYA5. Cells of each strain were mixed with phiYA5, and the bound phages were removed by centrifugation. Free phage titers were calculated as the ratio of the control (without cells). Cont. means control sample. Data are expressed as the mean with error bars representing ± SD (n=3). Bars with different letters are significantly different by Tukey's multiple comparison test.

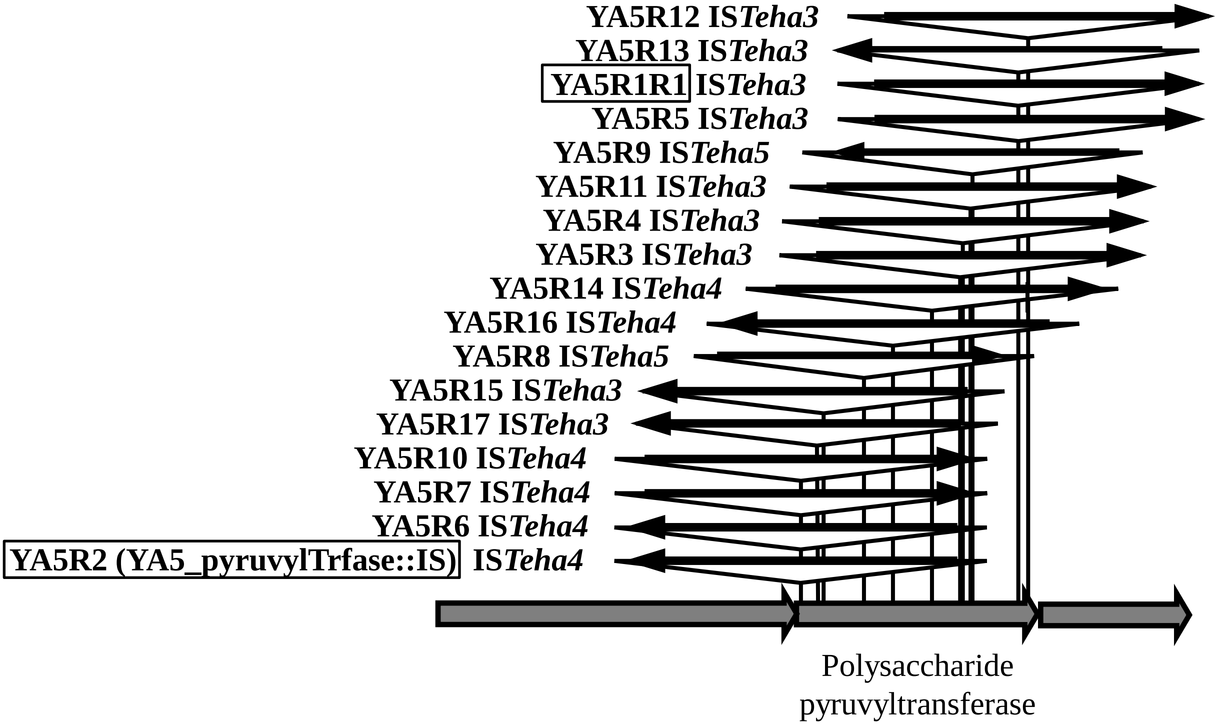

Figure S2: Schematic representation of the putative polysaccharide pyruvyltransferase gene and the location and orientation of ISs transposed in the derivatives of YA5.

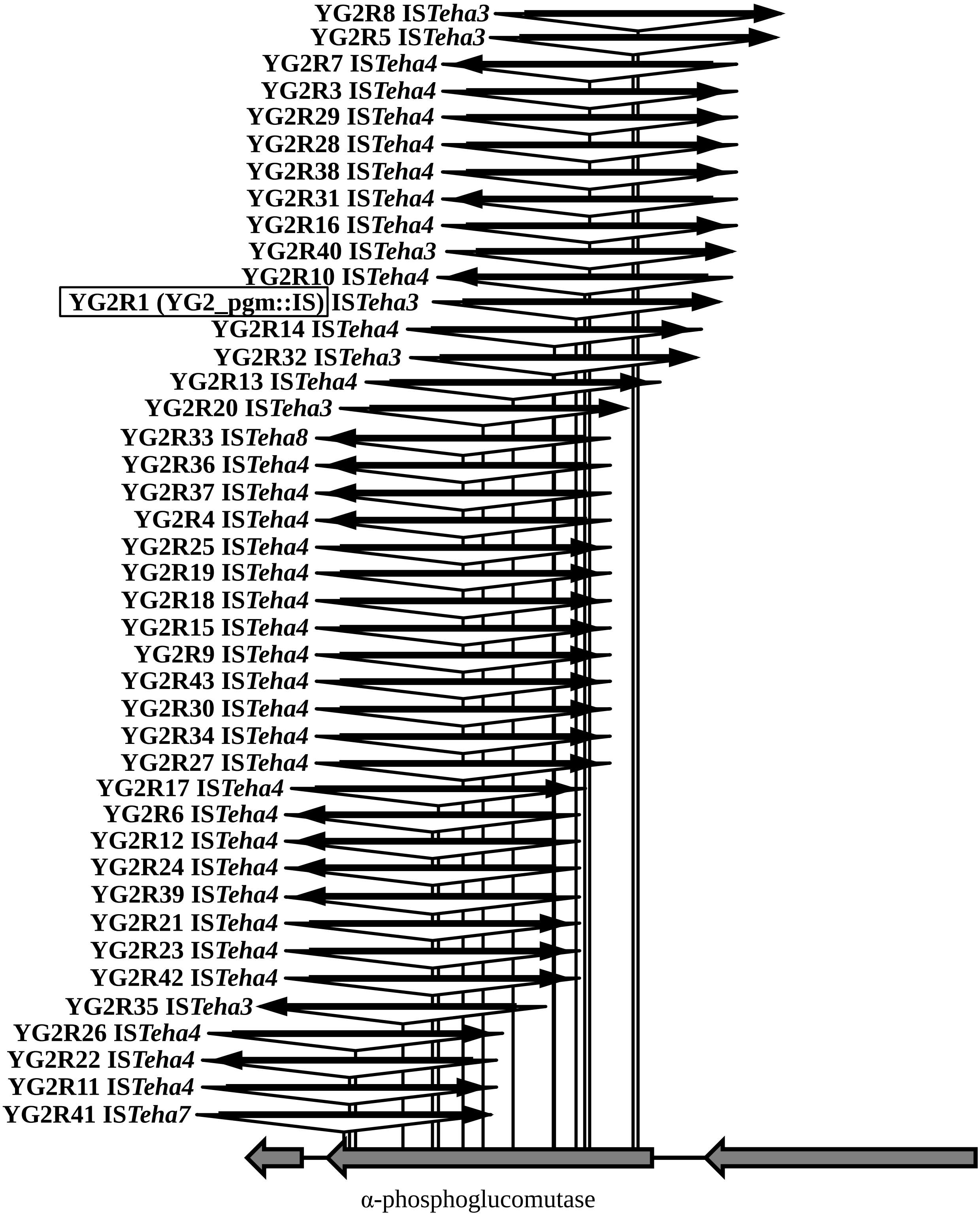

Figure S3: Schematic representation of the α-phosphoglucomutase gene and the location and orientation of ISs transposed in the derivatives of YG2.

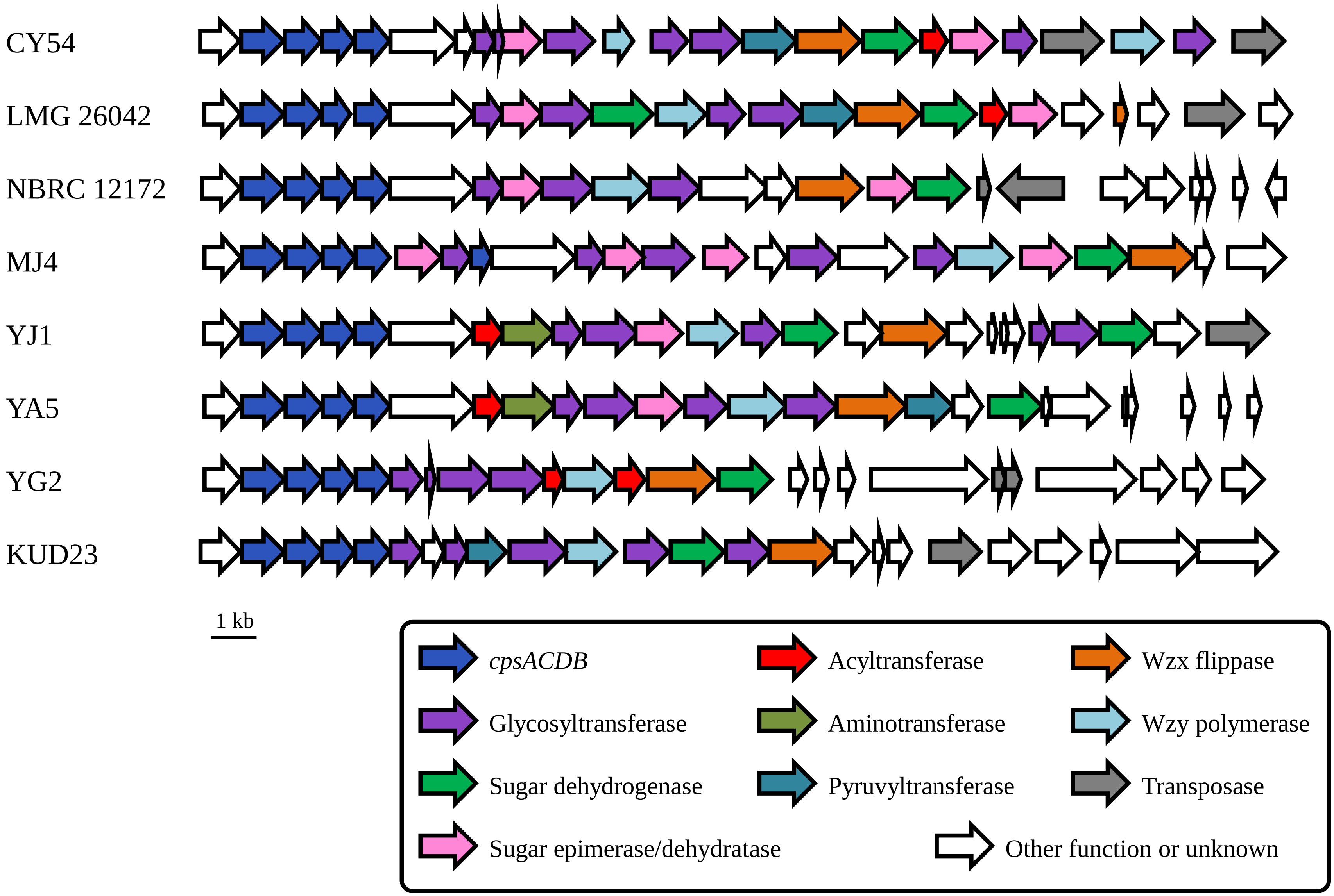

Figure S4: Comparison of *cps* loci among eight *T. halophilus* strains.

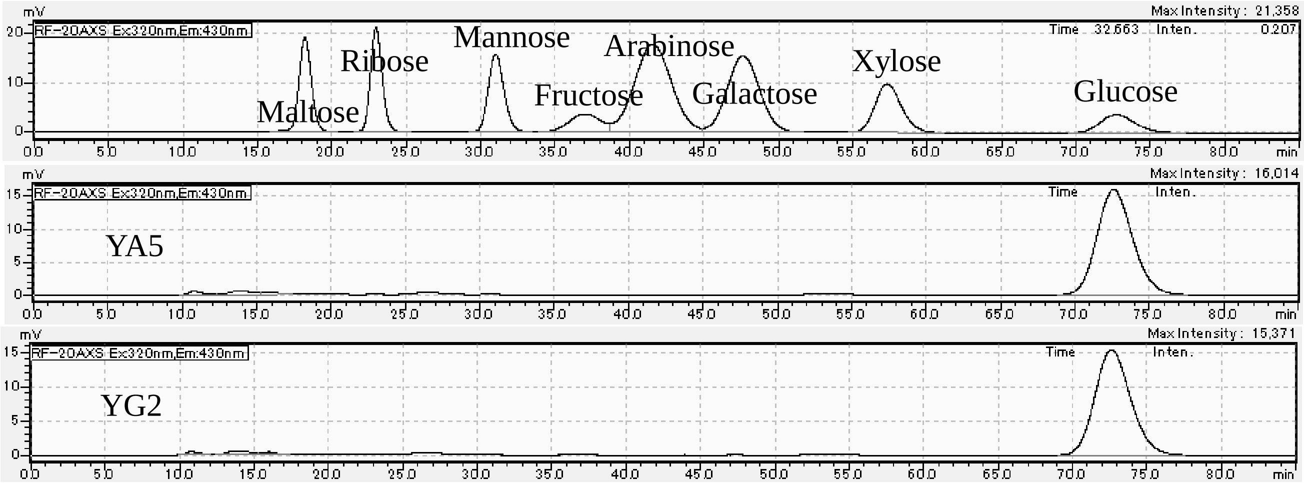

Figure S5: Sugar composition of CPSs from YA5 and YG2. Hydrolysates of CPS were analyzed with a reducing sugar analysis system.

A **IS*Teha7*** 1564 bp

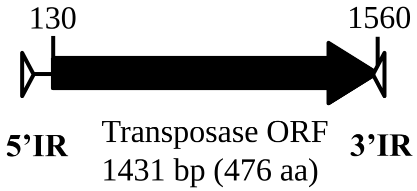


```
5'terminal  5'-ATGCCTACTGATTATAATTAATTTGTTGATTTACCAACATTTATTCAAAC-3'
               ||||||||||| ||    | |  | || |   |     |  ||      |
3'terminal  5'-ATGCCTACTGAATAATTGTGAACTTTTTAAAAAAGCTAAAATAAAGCGTC-3'
```

B **IS*Teha8*** 1563 bp

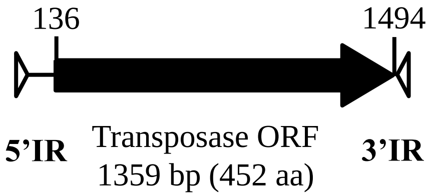


```
5'terminal  5'-CTAAAACTTGAAACACCGAAAACCCGACTAAAGGCTTGATATAACTAAGA-3'
               ||||||||||||| |   |||   |   | ||| ||             |
3'terminal  5'-CTAAAACTTGAAATATAAAAAGTACACATTAAGCCTATGGTATCAAGCAA-3'
```

Figure S6: Genetic structures and nucleotide sequences of the 5’ and 3’ termini of IS*Teha7* (A) and IS*Teha8* (B). The black arrows indicate ORFs that encode a putative transposase. The triangles indicate IRs at the 5’ and 3’ termini. The nucleotide numbers refer to the start and stop positions of the ORFs. Vertical lines between the sequences denote homologous nucleotides.

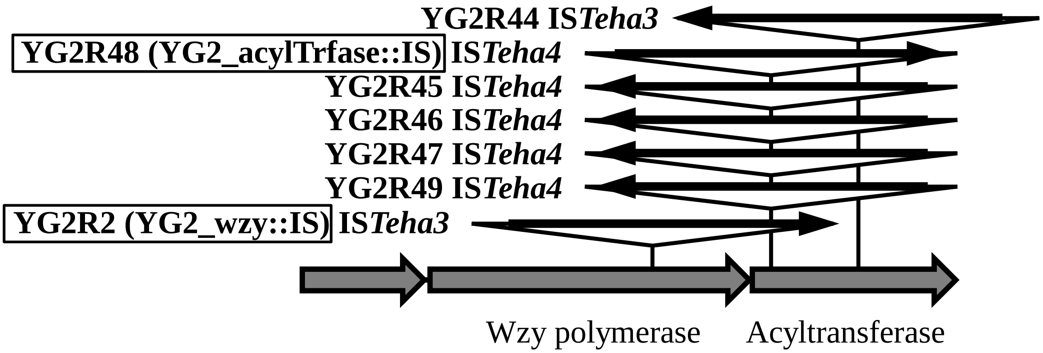

Figure S7: Schematic representation of Wzy polymerase and acyltransferase genes and the location and orientation of ISs transposed in the derivatives of YG2.

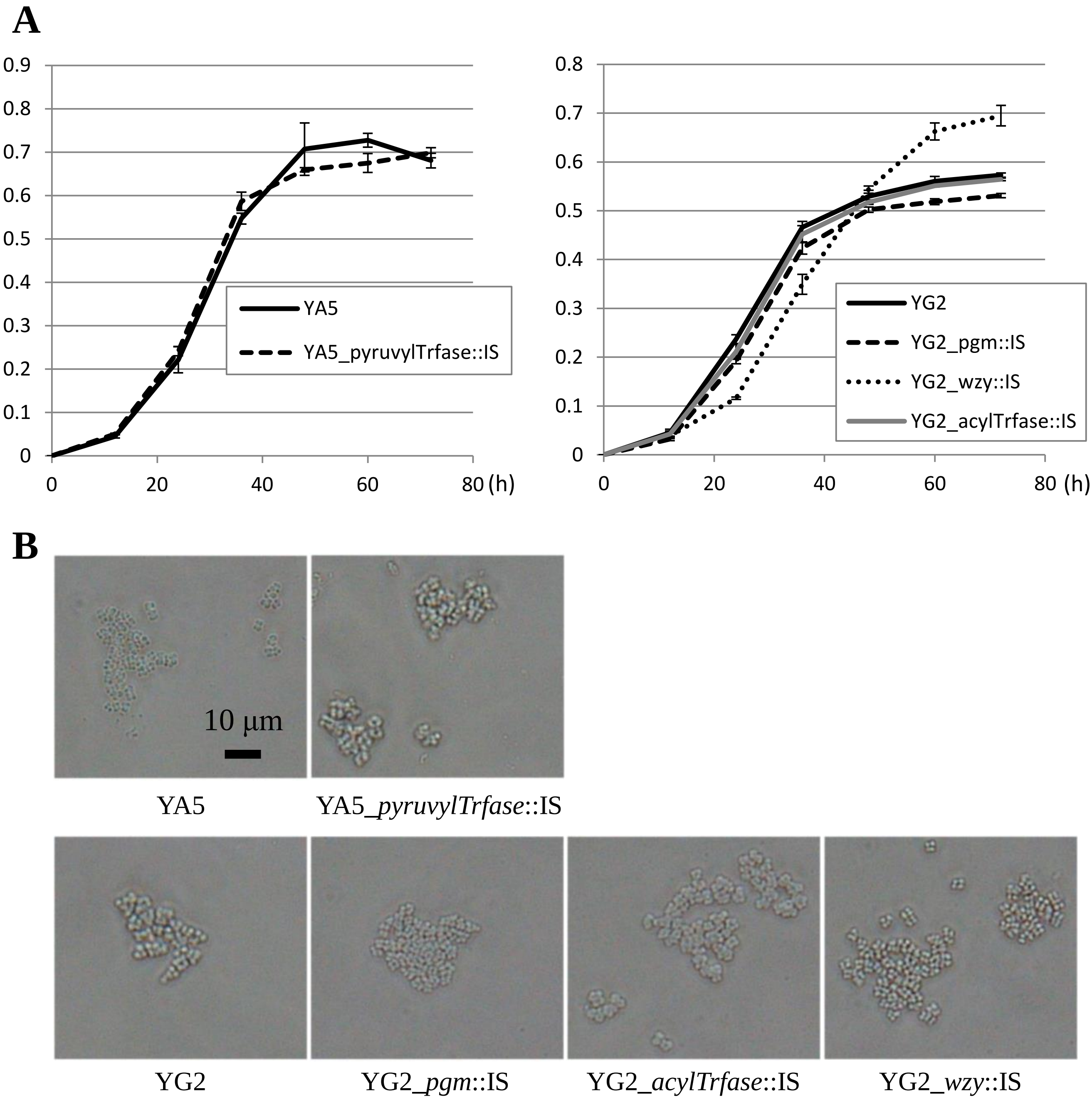

Figure S8: Growth and cell morphology of YA5, YG2 and their derivatives. (A) Growth curves. Fully grown cultures of each strain were 100-fold diluted in fresh MRS-10 medium, and the OD_{660} of the cultures was measured periodically. Data are expressed as the mean with error bars representing ± SD (n=3). (B) Cell morphology observed by light microscopy.

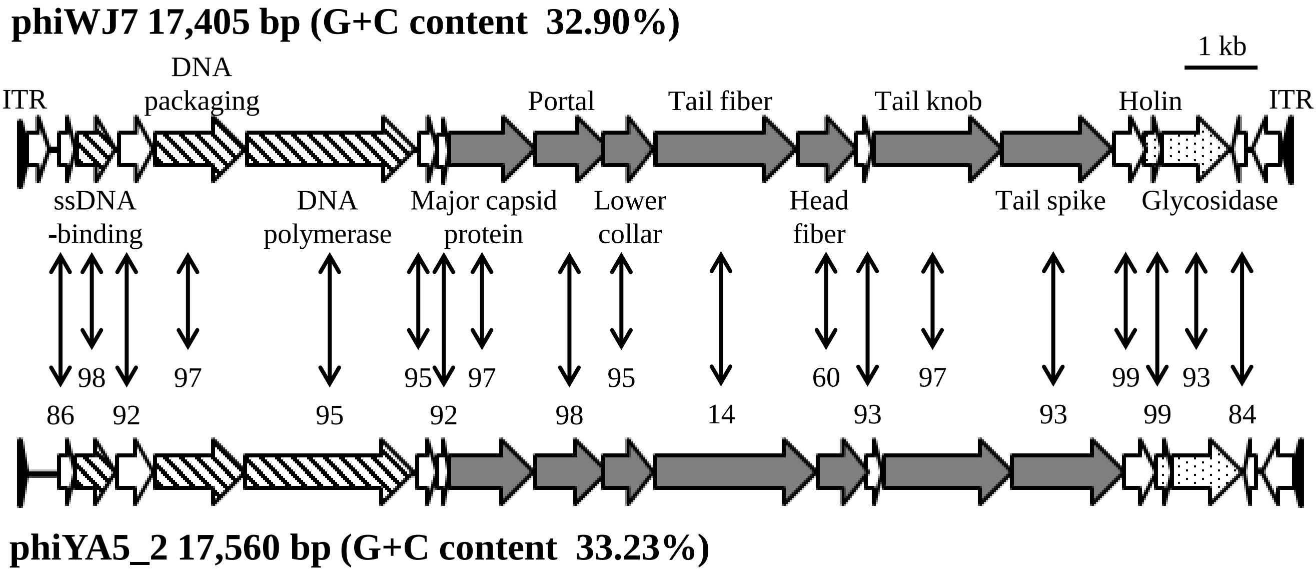

Figure S9: Genome comparison between phiWJ7 and phiYA5_2. Arrows indicate the possible ORFs. Functional groups are categorized into patterns (striped: DNA replication and packaging, gray-shaded: structural proteins, dotted: bacterial lysis, blanked: unknown). ITR indicates inverted terminal repeats and is marked by black triangles. The percentage shows the amino acid identities between phiWJ7 and phiYA5_2.
